# Association of Medicaid expansion with health insurance coverage by marital status and sex

**DOI:** 10.1371/journal.pone.0223556

**Published:** 2019-10-23

**Authors:** Jim P. Stimpson, Jessie Kemmick Pintor, Fernando A. Wilson

**Affiliations:** 1 Drexel University, Dornsife School of Public Health, Philadelphia, PA, United States of America; 2 University of Utah, Matheson Center for Health Care Studies, Salt Lake City, UT, United States of America; Duke University School of Medicine, UNITED STATES

## Abstract

**Objective:**

To determine the association of Medicaid expansion with health insurance coverage by marital status and sex.

**Methods:**

A population-based, quasi-experimental policy analysis was undertaken of the implementation of the Patient Protection and Affordable Care Act’s (ACA) Medicaid expansion provision on or after January 1, 2014. The 2010–16 American Community Survey provided data on 3,874,432 Medicaid-eligible adults aged 19–64 with incomes up to 138% of the federal poverty level. The outcome measures were no health insurance coverage and Medicaid coverage. The predictor variables were marital status and sex, with controls for family size, poverty status, race/ethnicity, education, employment status, immigration status, and metropolitan residence.

**Results:**

In 2016, the uninsured rate for married men and women in a Medicaid expansion state was 21.2% and 17.1%, respectively, compared to 37.4% for married men and 35.8% for married women in a non-expansion state. The Medicaid coverage rate grew between 14.8% to 19.3% in Medicaid expansion states, which contrasts with less than a 5% growth in non-expansion states. Triple differences analysis suggests that, for women of all age groups, Medicaid expansion resulted in a 1.6 percentage point lower uninsured rate for married women compared to unmarried women. For men, there was not a statistically significant difference in the uninsured rate for married compared to unmarried men. For women of all age groups, there was a 2.6 percentage point higher Medicaid coverage rate for married compared to unmarried women. For men, there was a 1.8 percentage point higher Medicaid coverage rate for married compared to unmarried men.

**Conclusion:**

Medicaid expansion under the ACA differentially lowered uninsurance and improved Medicaid coverage for married persons, especially married women, more than unmarried persons.

## Introduction

The Patient Protection and Affordable Care Act (ACA) expanded Medicaid coverage for adults up to 138 percent of the federal poverty level [1]. A Supreme Court decision made Medicaid expansion optional for states, rather than mandatory as originally intended. Consequently, 25 states had expanded Medicaid eligibility by January 1, 2014, which was the implementation date set by the ACA. More states have expanded since that date and there continues to be interest to expand Medicaid in states that have not yet passed legislation.

Since 2014, a growing evidence base on the impact of the ACA has indicated that the expansion of Medicaid not only has reduced the number of uninsured but has also improved access to care, utilization, affordability, and health outcomes [2–10]. The improved access to care has even been shown for vulnerable populations [11–14]. Moreover, there is evidence that Medicaid expansion has been associated with positive financial outcomes for states and hospitals [15–18].

However, there has been limited information on the impact of the ACA by marital status, which other research has shown to be an important predictor of health care access [19–20]. Married persons have higher rates of insurance coverage than formerly or never married persons, in part because a spouse can be covered by an employer’s health plan [21]. Marital disruption has been identified as a mechanism for differences in health insurance coverage [22]. For example, divorced and never married women are more likely to be uninsured compared to married women [23–25]. However, the specific effect of Medicaid expansion on Medicaid coverage by marital status is still unknown.

By extension, the effect of marital status on health insurance coverage has been found to vary by sex [26–27]. Although men have higher rates of uninsurance than women, women have much lower rates of employer-sponsored health insurance than men [21]. The ACA has reduced the uninsured population among women 19–44 years of age [28–29]. Lower incomes, on average, for women have been reported to be among the mechanisms for lower employer-sponsored insurance rates relative to men [21,26,30]. The reduced access to employer-sponsored coverage among women, particularly unmarried women, is troubling given the higher healthcare needs reported for women [29–30], and means that Medicaid expansion could be even more important for facilitating access to coverage for women to meet these healthcare needs.

In this study, we linked data on whether a state adopted Medicaid expansion with American Community Survey data and used a quasi-experimental approach to identify differences in health insurance coverage by marital status and level of implementation of Medicaid expansion in their state of residence. Based on prior research on insurance status [21–25], we hypothesized that married persons living in Medicaid expansion states would show greater improvement in Medicaid coverage compared to the same population living in states that chose not to expand Medicaid. Given that marital status is correlated with health insurance status and that the ACA reduced barriers for adults to qualify for Medicaid coverage, we hypothesized that the gains from Medicaid expansion would be greater for married persons compared to unmarried persons. Finally, we hypothesized that the gains from Medicaid expansion would be greater for women than for men.

## Methods

For our quasi-experimental study, we defined time as either as pre-ACA (2010–13) or post-ACA (2014–16). The treatment effect was defined by whether a state implemented Medicaid expansion after 2014. States that expanded Medicaid between January 1, 2014 to December 31, 2016 were classified as the treatment group, and states that did not expand Medicaid during the study period were classified as the control group. Persons included in the study were adults ages 19–64 with family incomes at or below 138 percent of the federal poverty level, which is consistent with prior studies of the ACA. We determined family poverty levels using the health insurance unit method, which defines a family on the basis of all individuals residing in a household who would be eligible for health insurance (either private or public) as a family unit, rather than including the income of all members of the household or using the Census family definition that includes all related family members within the household [31]. The Census family definition would aggregate, for example, the income of adult siblings residing in the same household, even though for insurance purposes they would be treated as separate units. There were no missing data in the database, so the final analytic sample size was 3,874,432 respondents. Because we used publicly available secondary data, this study was exempt from Institutional Review Board review. This study was not funded and therefore a funder did not play a role in this study.

We used the 2010–2016 American Community Survey (ACS) data provided by the Integrated Public Use Microdata Series [32]. The ACS is an annual survey conducted by the Census Bureau to collect social and economic characteristics of persons and households and includes the Federal Information Processing Standards (FIPS) code, which permits the data to be linked with other databases at the state and county level. Data about state decisions on Medicaid expansion came from the Henry J. Kaiser Family Foundation’s State Health Facts database including the date of implementation [33]. We added the state FIPS codes to the Kaiser database and then linked it to the ACS data using the state identifier. The combined databases enabled the analysis of individual outcomes for persons living in a state that implemented Medicaid expansion.

The outcome variable was whether the respondent had health insurance coverage or Medicaid coverage. The predictor variable was marital status, defined as married versus unmarried. Analyses were stratified by sex (female or male) and age group (19–25, 26–44, or 45–64 years). We classified the first age group as 19–25 because the ACA allows someone to be covered by their parent’s insurance plan until age 26. Control variables included age in years, number of children (three or more vs. less than three), poverty status (income of 0–100 percent versus 101–138 percent of the federal poverty level), race/ethnicity (non-Hispanic White, Hispanic, non-Hispanic Black, or other race/ethnicity), education (less than high school, high school, some college, or college), employment status (unemployed or employed), immigration status (US born, naturalized citizen, not a citizen), and residence in a metropolitan area.

All analyses were conducted using Stata/MP, version 15 and accounted for survey weights and robust standard errors. Statistical significance was assumed at p-values of less than 0.05. Survey weighted characteristics of the sample were calculated and stratified by state Medicaid expansion status and marital status. Likewise, survey weighted percentages of persons without health insurance or covered by Medicaid insurance were calculated for each year and stratified by state Medicaid expansion status, marital status, and sex.

We used a triple-differences (difference-in-difference-in-difference) approach to compare the pre- and post-ACA trend in the outcomes (uninsured and Medicaid coverage) by marital status and sex between expansion and non-expansion states. This method isolates the change in insurance coverage in the study period that resulted from Medicaid expansion compared to what the coverage rate would have been if the state had not expanded Medicaid. Our triple-differences analysis measured the impact of Medicaid expansion on the percentage of persons without health insurance or covered by Medicaid through a three-way interaction effect by population group (marital status), time (pre- and post-ACA period), and treatment (state Medicaid expansion status). We also stratified the analysis by sex and age group. For ease of interpretation and to enable comparison with other ACA studies, we estimated linear probability regression models adjusted for state and year fixed effects. Data shown in the S1 Fig suggest that the parallel trends assumption for triple-differences analysis was met through the regression estimator and a visual inspection of the line graphs. We conducted sensitivity analyses that are available in the supporting information. First, we re-analyzed the triple-differences linear probability models after dropping states that expanded Medicaid eligibility before January 2014 or expanded late in either 2015 or 2016.

There are several limitations to consider for interpreting the results of this study. First, our estimates of insurance coverage are measured in annual increments that are based on the respondent’s insurance coverage at the time of the survey and thus are not sensitive to coverage variations in a given year [34]. For example, a respondent may have started a year with employer-based coverage, then lost that coverage and then subsequently later in the year gained coverage through Medicaid expansion, but the ACS data would only pick up the type of insurance coverage they had at the time of the survey. However, we do not expect this to bias the results and federal surveys that use annual estimates are regarded as the definitive source of health insurance coverage [20–21, 35]. This limitation also applies to our measure of marital status, which could have varied within a year. Although we adjusted for a range of potential confounders at the individual level that were available in the ACS, it is possible that there were control variables inadvertently omitted that could possibly attenuate the results. Related, there are many other state policies that could affect Medicaid coverage such as state Medicaid waivers (e.g. section 1115) or income eligibility limits that were not measured in this study and were assumed to be held constant by our state and year fixed effects triple differences analysis.

## Results

Table 1 shows the survey weighted sample characteristics for persons eligible for Medicaid stratified by marital status and Medicaid expansion status. Among both unmarried and married persons, most characteristics were similar regardless of state Medicaid expansion status. However, compared to persons residing in a non-expansion state, persons who resided in a state that expanded Medicaid were more likely to report ‘other’ race/ethnicity (9.7% vs 5.5% for unmarried persons and 14.1% vs 6.9% for married) or have a residence in a metropolitan area (81.4% vs 71.5% for unmarried and 79.9% vs 69.3% for married) and were less likely to be non-Hispanic Black (17.0% vs 26.3% for unmarried and 8.6% vs 13.7% for married).

**Table 1:** Percentage of Medicaid eligible respondents by marital status and state Medicaid expansion status, American Community Survey 2010–16, N = 3,874,432.

	Not Married		Married	
	No Expansion	Expansion	No Expansion	Expansion
	N = 1,139,589	N = 1,850,138	N = 364,513	N = 520,192
Sex, %				
Female	52.5%	51.8%	51.7	51.9
Male	47.5%	48.2%	48.3	48.1
Age, years, %				
19–25	41.3%	43.3%	10.4	8.8
26–44	33.5%	32.4%	51.5	50.6
45–64	25.2%	24.3%	38.1	40.6
Number of Children, %				
0	75.4	77.3	33.1	31.3
1	11.8	10.8	19.2	19.4
2	7.3	6.7	22.0	22.3
3 or more	5.4	5.1	25.7	27.0
Federal Poverty Level, %				
Income 100–138%	19.2	18.3	36.0	35.1
Income <100%	80.8	81.7	64.0	64.9
Race/ethnicity, %				
Non-Hispanic White	50.0	52.0	45.5	43.3
Hispanic	18.2	21.3	33.9	34.0
Non-Hispanic Black	26.3	17.0	13.7	8.6
Other	5.5	9.7	6.9	14.1
Immigration Status, %				
Born in the United States	88.2	85.2	66.1	56.8
Naturalized Citizen	3.2	4.7	7.9	13.2
Non-Citizen	8.6	10.1	26.0	30.0
Education, %				
Less than high school	17.2	15.7	27.8	28.1
High school	43.8	41.9	42.1	41.3
Some college	30.0	31.3	19.5	18.7
College	9.0	11.1	10.6	11.9
Employment Status, %				
Not employed	43.0	41.8	45.0	43.8
Employed	57.0	58.2	55.0	56.2
Metropolitan Residence, %				
Not metro	28.5	18.6	30.7	20.1
Metro	71.5	81.4	69.3	79.9

NOTE: Percentages are based on the sample weights provided by the Census Bureau. Medicaid eligibility is defined by age (19–64) and income (below 139% of the federal poverty level).

Table 2 shows the weighted percentage of Medicaid eligible persons from 2010 to 2016 reporting no health insurance or Medicaid coverage stratified by marital status, sex, and state Medicaid expansion implementation. The percentage of persons with Medicaid coverage increased from 2010 to 2016 for all groups. Persons residing in a Medicaid expansion state were less likely to report no health insurance coverage compared to persons that did not live in an expansion state. The uninsured rate decreased about 20% in Medicaid expansion states and decreased 14% in non-expansion states. In 2016, the uninsured rate for married men and women in a Medicaid expansion state was 21.2% and 17.1%, respectively, compared to 37.4% for married men and 35.8% for married women in a non-expansion state. In 2016, the uninsured rate for unmarried men and women in a Medicaid expansion state was 19.4% and 12%, respectively, compared to 35.3% for men and 26.8% for women in a non-expansion state.

**Table 2:** Trend in no health insurance coverage and Medicaid coverage by marital status, sex, and state Medicaid expansion status, American Community Survey 2010–16, N = 3,874,432 Medicaid eligible respondents.

	**No Health Insurance Coverage**											
	**Non-Medicaid Expansion States**						**Medicaid Expansion States**					
** **	**Overall**		**Men**		**Women**		**Overall**		**Men**		**Women**	
** **	**Married**	**Not Married**	**Married**	**Not Married**	**Married**	**Not Married**	**Married**	**Not Married**	**Married**	**Not Married**	**Married**	**Not Married**
2010	50.8%	45.1%	51.8%	51.0%	49.8%	39.7%	40.2%	36.2%	42.4%	42.7%	38.2%	30.0%
2011	50.6%	43.2%	52.1%	48.5%	49.3%	38.4%	39.4%	34.1%	41.4%	39.8%	37.5%	28.7%
2012	49.1%	41.9%	50.0%	46.6%	48.3%	37.6%	38.9%	32.8%	40.9%	38.3%	37.0%	27.7%
2013	47.6%	40.9%	48.3%	45.4%	47.0%	36.8%	36.9%	31.9%	38.7%	36.8%	35.2%	27.2%
2014	42.4%	36.0%	43.0%	40.6%	41.9%	31.9%	27.9%	24.3%	29.8%	29.1%	26.1%	19.9%
2015	37.7%	32.4%	39.0%	37.2%	36.5%	28.2%	21.4%	18.0%	23.4%	22.2%	19.5%	14.1%
2016	36.6%	30.8%	37.4%	35.3%	35.8%	26.8%	19.1%	15.6%	21.2%	19.4%	17.1%	12.0%
Difference, 2016–2010	-14.2%	-14.3%	-14.4%	-15.7%	-14.0%	-12.9%	-21.2%	-20.6%	-21.2%	-23.3%	-21.1%	-18.1%
	**Medicaid Coverage**											
	**Non-Medicaid Expansion States**						**Medicaid Expansion States**					
** **	**Overall**		**Men**		**Women**		**Overall**		**Men**		**Women**	
** **	**Married**	**Not Married**	**Married**	**Not Married**	**Married**	**Not Married**	**Married**	**Not Married**	**Married**	**Not Married**	**Married**	**Not Married**
2010	19.0%	21.0%	18.2%	15.8%	19.7%	25.7%	30.4%	28.2%	28.5%	21.8%	32.1%	34.1%
2011	19.6%	21.0%	18.8%	16.0%	20.4%	25.7%	32.0%	28.1%	30.5%	22.0%	33.5%	33.9%
2012	20.4%	21.5%	19.4%	16.5%	21.3%	26.0%	32.3%	28.5%	30.5%	22.5%	33.9%	34.1%
2013	20.2%	21.4%	19.6%	16.6%	20.8%	25.8%	32.7%	29.1%	31.0%	23.8%	34.3%	34.1%
2014	21.5%	22.5%	20.8%	17.6%	22.3%	26.9%	40.0%	34.7%	38.3%	28.9%	41.5%	40.1%
2015	22.8%	23.5%	22.0%	18.6%	23.5%	27.8%	47.2%	40.6%	45.4%	35.5%	48.8%	45.4%
2016	23.2%	24.2%	22.5%	19.5%	23.9%	28.3%	49.6%	42.9%	47.8%	37.9%	51.3%	47.6%
Difference, 2016–2010	4.3%	3.2%	4.3%	3.7%	4.1%	2.6%	19.3%	14.8%	19.2%	16.1%	19.2%	13.5%

NOTE: Percentages are based on the sample weights provided by the Census Bureau. Medicaid eligibility is defined by age (19–64) and income (below 139% of the federal poverty level).

The second panel in Table 2 indicates that persons residing in a Medicaid expansion state were more likely to report Medicaid coverage compared to persons that did not live in an expansion state. The Medicaid coverage rate grew between 14.8% to 19.3% in Medicaid expansion states, which contrasts with less than a 5% growth in non-expansion states. In 2016, the Medicaid coverage rate for married men and women in a Medicaid expansion state was 47.8% and 51.3%, respectively, compared to 22.5% for married men and 23.9% for married women in a non-expansion state. In 2016, the Medicaid coverage rate for unmarried men and women in a Medicaid expansion state was 37.9% and 47.6%, respectively, compared to 19.5% for men and 28.3% for women in a non-expansion state.

Table 3 provides the results for the triple differences linear probability regression for Medicaid eligible respondents adjusted for state and year fixed effects. For women of all age groups, there was a 1.6 percentage point lower uninsured rate for married compared to unmarried women, resulting from residing in a Medicaid expansion state pre- vs. post-ACA. For men, there was not a statistically significant difference in the uninsured rate for married compared to unmarried men. We also stratified the analyses by age groups to isolate the effect of marital status and sex at different points in the life course. Married women ages 19–25 had a statistically significant lower uninsured rate (-3.1%) compared to unmarried women; however, the results for women age 26–64 were not statistically significant. Married men age 19–25 had a lower uninsured rate (-3.1%) compared to unmarried men that was statistically significant, while married men age 26–44 (3.6%) and 45–64 (1.6%) had a higher uninsured rate compared to unmarried men as a result of Medicaid expansion.

**Table 3:** Triple differences linear probability model for no health insurance coverage by marital status, sex, age, and state Medicaid expansion status, American Community Survey 2010–16.

	Women				Men			
	All Ages	Age 19–25	Age 26–44	Age 45–64	All Ages	Age 19–25	Age 26–44	Age 45–64
**Before 2014**								
Control–Not married & No State Medicaid Expansion	0.354	0.357	0.542	0.310	0.437	0.461	0.609	0.347
Control–Married & No State Medicaid Expansion	0.394	0.435	0.546	0.353	0.393	0.455	0.534	0.321
Treated–Not Married & Medicaid Expansion State	0.143	0.220	0.248	0.295	0.236	0.304	0.425	0.136
Treated–Married & Medicaid Expansion State	0.159	0.250	0.245	0.328	0.172	0.329	0.521	0.109
**Difference for 2010–2013**								
Coefficient	-0.024	-0.048	-0.007	-0.011	-0.020	-0.019	-0.021	-0.002
Standard Error	0.003	0.007	0.004	0.004	0.003	0.009	0.004	0.005
t	9.06	6.72	1.74	2.38	7.20	2.17	5.07	0.53
p-value	<0.001	<0.001	0.081	0.017	<0.001	0.030	<0.001	0.598
**After 2014**								
Control–Not married & No State Medicaid Expansion	0.233	0.221	0.399	0.191	0.278	0.297	0.454	0.253
Control–Married & No State Medicaid Expansion	0.263	0.281	0.412	0.209	0.230	0.290	0.388	0.191
Treated–Not Married & Medicaid Expansion State	-0.015	0.064	0.065	0.117	0.019	0.132	0.281	-0.027
Treated–Married & Medicaid Expansion State	-0.025	0.044	0.066	0.120	-0.043	0.074	0.229	-0.075
**Difference for 2014–2016**								
Coefficient	-0.040	-0.079	-0.011	-0.016	-0.013	-0.051	0.015	0.014
Standard Error	0.003	0.008	0.005	0.005	0.003	0.010	0.005	0.005
t	13.59	9.38	2.37	3.27	4.26	4.85	3.00	2.72
p-value	<0.001	<0.001	0.018	0.001	<0.001	<0.001	0.003	0.006
**Triple Difference**								
Coefficient	-0.016	-0.031	-0.004	-0.005	0.007	-0.031	0.036	0.016
Standard Error	0.004	0.011	0.006	0.007	0.004	0.014	0.007	0.007
t	-3.94	-2.79	-0.60	-0.75	1.58	-2.29	5.58	2.37
p-value	<0.001	0.005	0.551	0.455	0.115	0.022	<0.001	0.018
R-square	0.13	0.13	0.16	0.11	0.14	0.18	0.14	0.11
N	1,972,877	659,774	685,631	627,472	1,901,555	665,282	662,739	573,534

NOTE: Estimates are based on the sample weights provided by the Census Bureau and adjusted for state and year fixed effects. Medicaid eligibility was defined by age 19–64 and income below 139% of the federal poverty level. Multivariate adjustment included the following control variables: age, number of children, race/ethnicity, immigration status, poverty status, education, employment status, and metropolitan residence.

Table 4 provides the results for the triple differences linear probability regression for Medicaid eligible respondents adjusted for state and year fixed effects. For women of all age groups, there was a 2.6 percentage point higher Medicaid coverage rate for married compared to unmarried women, resulting from Medicaid expansion pre- vs. post-ACA. For men, there was a 1.8 percentage point higher Medicaid coverage rate for married compared to unmarried men. Married women ages 26–44 had a statistically significant higher Medicaid coverage rate compared to unmarried women; however, the results for women age 19–25 and 45–64 were not statistically significant. Married men age 26–44 had a lower Medicaid coverage rate compared to unmarried men that was statistically significant, while married men age 45–64 had a statistically significantly a higher Medicaid coverage rate compared to unmarried men. There was not a statistically significant relationship for men age 19–25.

**Table 4:** Triple differences linear probability model for Medicaid coverage by marital status, sex, age, and state Medicaid expansion status, American Community Survey 2010–16.

	Women				Men			
	All Ages	Age 19–25	Age 26–44	Age 45–64	All Ages	Age 19–25	Age 26–44	Age 45–64
**Before 2014**								
Control–Not married & No State Medicaid Expansion	0.055	0.135	0.058	0.175	0.010	0.083	0.072	0.117
Control–Married & No State Medicaid Expansion	-0.059	0.071	-0.022	0.022	-0.014	0.121	0.102	0.540
Treated–Not Married & Medicaid Expansion State	0.357	0.302	0.512	0.175	0.258	0.198	0.136	0.461
Treated–Married & Medicaid Expansion State	0.273	0.317	0.439	0.029	0.268	0.287	0.197	0.398
**Difference for 2010–2013**								
Coefficient	0.029	0.08	0.007	0.007	0.034	0.051	0.032	0.001
Standard Error	0.002	0.007	0.004	0.004	0.002	0.007	0.004	0.004
t	12.13	11.48	1.82	1.87	14.85	6.87	9.04	0.18
p-value	<0.001	<0.001	0.069	0.061	<0.001	<0.001	<0.001	0.854
**After 2014**								
Control–Not married & No State Medicaid Expansion	0.099	0.160	0.136	0.244	0.075	0.118	0.144	0.181
Control–Married & No State Medicaid Expansion	-0.010	0.122	0.052	0.097	0.053	0.163	0.172	0.120
Treated–Not Married & Medicaid Expansion State	0.482	0.382	0.678	0.359	0.413	0.295	0.325	0.624
Treated–Married & Medicaid Expansion State	0.429	0.443	0.617	0.229	0.443	0.413	0.369	0.582
**Difference for 2014–2016**								
Coefficient	0.055	0.098	0.022	0.017	0.052	0.073	0.015	0.018
Standard Error	0.003	0.009	0.005	0.005	0.003	0.010	0.005	0.005
t	18.20	10.92	4.77	3.60	17.53	7.20	3.40	3.55
p-value	<0.001	<0.001	<0.001	<0.001	<0.001	<0.001	0.001	<0.001
**Triple Difference**								
Coefficient	0.026	0.019	0.015	0.010	0.018	0.022	-0.016	0.017
Standard Error	0.004	0.011	0.006	0.006	0.004	0.013	0.006	0.006
t	6.62	1.66	2.57	1.63	4.81	1.76	-2.85	2.67
p-value	<0.001	0.097	0.010	0.104	<0.001	0.078	0.004	0.008
R-square	0.18	0.21	0.18	0.16	0.15	0.11	0.14	0.13
N	1,972,877	659,774	685,631	627,472	1,901,555	665,282	662,739	573,534

NOTE: Estimates are based on the sample weights provided by the Census Bureau and adjusted for state and year fixed effects. Medicaid eligibility was defined by age 19–64 and income below 139% of the federal poverty level. Multivariate adjustment included the following control variables: age, number of children, race/ethnicity, immigration status, poverty status, education, employment status, and metropolitan residence.

The sensitivity tests, shown in the S1 Table, suggest the results in the triple differences models are robust to model specification. We re-analyzed the triple difference models after dropping states that expanded Medicaid earlier than January 2014 or later in 2015 or 2016 to isolate differences in state implementation of Medicaid expansion. We found that the estimates were similar suggesting that the results are robust to the timing of the Medicaid expansion.

## Discussion

Our study joins a large and growing body of literature that demonstrates that persons living in Medicaid expansion states experienced improved health insurance coverage compared to the same population living in states that chose not to expand Medicaid [2–3]. As more studies using rigorous policy analysis designs are added to the research literature, our confidence in the causal relationship grows that Medicaid expansion has been responsible, in part, for the reduction in persons without health insurance [2–3]. The evidence from these policy analyses should be considered by states that have not expanded Medicaid yet or states that might be considering eliminating expansion or implementing a reduced version of expansion [36].

Our primary aim was to examine if the effect of Medicaid expansion varied by marital status and sex. The results of our quasi-experimental analysis and subsequent sensitivity analyses suggest that the gains in health insurance coverage from Medicaid expansion were greater for married persons compared to unmarried persons among all age groups [14]. The differential effect in favor of married persons is likely related to findings in the literature that have found that persons who experience marital disruption have lower odds of being insured [22–25]. Possibly marital disruption complicates the eligibility criteria and Medicaid application for formerly married persons [22,24]. Unmarried persons may have limited time to apply for benefits or lower awareness of eligibility for benefits [37]. Unmarried persons have historically been more likely to be uninsured than their married counterparts and it appears these differences have widened post-ACA.

Adults without minor children were the primary focus of eligibility changes under the Medicaid expansion. Therefore, one possible explanation for any difference by marital status in changes in Medicaid coverage could be differences in the presence of minor children in the household. However, we found that unmarried persons were far less likely to have minor children, so we would expect that the increase would have been greater among these individuals than among married persons, which was not the case. Evidence of the “welcome-mat effect,” whereby individuals who were previously (pre-ACA) eligible but unenrolled gained Medicaid coverage after full implementation of the ACA in 2014 [38], may help explain our findings, as married persons may have been more likely to be eligible for Medicaid pre-ACA given their higher likelihood of having minor children.

Consistent with prior literature on sex differences in health insurance, we found that evidence for sex differences in the change in health insurance coverage from Medicaid expansion by marital status [28–30]. Women may have more favorable Medicaid enrollment from state Medicaid expansion in part from higher need for coverage than men [21] and due to worse access to employer-sponsored coverage and lower incomes than men [21,26,30]. Given the higher healthcare needs reported for women [29–30], a differential uptake in Medicaid may help to mitigate disparities in employer-sponsored insurance coverage between men and women. Medicaid expansion has also served an important role in ensuring continuity of coverage for women who previously may have only had access to Medicaid during pregnancy. Because eligibility levels for prenatal coverage differ from overall adult eligibility, oftentimes women are only able to access Medicaid for the duration of pregnancy plus 60 days postpartum, restricting them from receiving health care services at any other time [28–29]. One important goal of the ACA Medicaid expansion was to bring consistency and continuity to Medicaid eligibility across states and hence provide more equitable access in the face of gaps, such as those faced by women of reproductive age. We provide more evidence that the lack of implementation of Medicaid expansion disproportionately impacts unmarried men and women in states that chose not to expand Medicaid, who may have already been at higher risk for uninsurance given previous wide variation in state-level Medicaid policy [27].

Our analyses were also stratified by age groups, and we found that age, at times, attenuated or altered the relationship between Medicaid expansion, marital status, and sex. In part, this difference may be driven by life course differences in access to health insurance. The ACA allows persons to remain on their parent’s health insurance up to age 26, which provides a hedge against economic and job insecurity during at younger ages. Likewise, those in younger age groups may disproportionately benefit from the policies in the ACA that mandated employers to offer coverage and by the state health insurance exchanges established to offer coverage outside of one’s employer [14]. Finally, age group variations can also be driven by changes in marital status as persons in younger age groups are more likely to be never married, and older age groups are more likely to be married or previously married, and these differences in marital status have been found to be associated with variations in insurance coverage in correlational and cohort studies [21–25].

## Supporting information

S1 Fig Parallel trends test. (DOCX)

S1 Table Sensitivity test that excludes early and late expanders. (DOCX)
