# Supplementary material for: Association of Medicaid expansion with health insurance coverage by marital status and sex
Source: PLoS One. 2019 Oct 23;14(10):e0223556. doi: 10.1371/journal.pone.0223556 (PMC6808332; doi:10.1371/journal.pone.0223556)
Supplement: S1 Fig — (DOCX) [file pone.0223556.s001.docx]

**S1 Fig: Parallel Trends Test**

First, we visually inspected the trend lines for expansion and non-expansion states by sex and marital status from the 2010-2016 American Community Survey data and determined the lines are parallel. Second, we calculated the coefficients on the triple interaction and then calculated a joint F-test across the coefficients for the pre-expansion period (2010-2013).

The following is a summary of the chi square tests:

|  | **Uninsured** | | **Medicaid** | |
| --- | --- | --- | --- | --- |
|  | Chi square | p-value | Chi square | p-value |
| Year x Expansion State x Marital Status | 82.80 | < 0.000 | 39.96 | < 0.000 |
| Year x Marital Status | 31.44 | < 0.000 | 8.26 | 0.041 |
| Year x Medicaid Expansion | 1.51 | 0.679 | 4.60 | 0.204 |
