# Supplementary material for: Association of Medicaid expansion with health insurance coverage by marital status and sex
Source: PLoS One. 2019 Oct 23;14(10):e0223556. doi: 10.1371/journal.pone.0223556 (PMC6808332; doi:10.1371/journal.pone.0223556)
Supplement: S1 Table — (DOCX) [file pone.0223556.s002.docx]

**S1 Table: Sensitivity Test That Excludes Early and Late Expanders**. Triple Differences Linear Probability Model for No Health Insurance and Medicaid Coverage by Marital Status and State Medicaid Expansion Status, Excluding States that Expanded Medicaid Early or Late, American Community Survey 2010–16

|  | **Uninsured** | | **Medicaid** | |
| --- | --- | --- | --- | --- |
| **Before 2014** | No Exclusion | Exclude Early and Late Expanders | No Exclusion | Exclude Early and Late Expanders |
| Control - No State Medicaid Expansion * Not married | 0.364 | 0.363 | 0.057 | 0.067 |
| Control - No State Medicaid Expansion * Married | 0.368 | 0.363 | -0.023 | -0.009 |
| Treated - Medicaid Expansion State * Not married | 0.159 | 0.294 | 0.331 | 0.101 |
| Treated - Medicaid Expansion State * Married | 0.141 | 0.268 | 0.284 | 0.067 |
| Difference for 2010-2013 |  |  |  |  |
| Coefficient | -0.022 | -0.026 | 0.032 | 0.042 |
| Standard Error | 0.002 | 0.002 | 0.002 | 0.002 |
| t | 11.38 | 11.52 | 19.05 | 20.57 |
| p-value | <0.001 | <0.001 | <0.001 | <0.001 |
| **After 2014** |  |  |  |  |
| Control - No State Medicaid Expansion * Not married | 0.236 | 0.218 | 0.102 | 0.123 |
| Control - No State Medicaid Expansion * Married | 0.233 | 0.211 | 0.026 | 0.051 |
| Treated - Medicaid Expansion State * Not married | -0.016 | 0.106 | 0.462 | 0.242 |
| Treated - Medicaid Expansion State * Married | -0.047 | 0.078 | 0.441 | 0.229 |
| Difference for 2014-2016 |  |  |  |  |
| Coefficient | -0.027 | -0.021 | 0.054 | 0.059 |
| Standard Error | 0.002 | 0.002 | 0.002 | 0.003 |
| t | 12.58 | 8.49 | 25.37 | 23.06 |
| p-value | <0.001 | <0.001 | <0.001 | <0.001 |
| **Triple Difference** |  |  |  |  |
| Coefficient | -0.005 | 0.005 | 0.022 | 0.017 |
| Standard Error | 0.003 | 0.003 | 0.003 | 0.003 |
| t | -1.77 | 1.57 | 8.14 | 5.30 |
| p-value | 0.077 | 0.116 | <0.001 | <0.001 |
| R-square | 0.14 | 0.14 | 0.17 | 0.18 |
| N | 3,874,432 | 2,783,130 | 3,874,432 | 2,783,130 |

NOTE: Estimates are based on the sample weights provided by the Census Bureau and adjusted for state and year fixed effects. Medicaid eligibility was defined by age 19-64 and income below 139% of the federal poverty level. Multivariate adjustment included the following control variables: age, sex, number of children, race/ethnicity, immigration status, poverty status, education, employment status, and metropolitan residence. Early expanders include California, Connecticut, DC, Minnesota, New Jersey, Washington. Late expanders include Alaska, Indiana, Louisiana, Montana, Pennsylvania.
